# UNDERSTANDING PERSON-CENTERED CARE FOR OLDER PEOPLE IN SIX DEVELOPING COUNTRIES

**DOI:** 10.1093/geroni/igad104.0969

**Published:** 2023-12-21

**Authors:** Jing Wang, Barbara Bowers

**Affiliations:** University of New Hampshire, Durham, New Hampshire, United States; University of Wisconsin-madison, Madison, Wisconsin, United States

## Abstract

Person-centered care (PCC) is a widely acknowledged and encouraged approach to quality care for older adults residing in residential care facilities. However, current understandings of PCC are mainly based on research and practice in western, first world nations. The lack of research on PCC in developing countries leads to uncertainty over whether and how current understandings of PCC apply to cultures and contexts in non-western, developing countries. To enhance care and outcomes for diverse populations, it is crucial to comprehend and culturally adapt PCC understandings and approaches. Therefore, we conducted interviews with researchers and practitioners from six countries in various stages of development to identify elements that form the core of high-quality care for older people and how they relate to Western notions of PCC. We gained valuable insights into how cultural differences, population health priorities, political conflict, and limited resources impact their understanding and implementation of PCC. In addition to variations in the language of PCC, some first world assumptions about PCC do not resonate well in developing countries. One example is the emphasis on individualism, a basic element of PCC. The role of the older person in decision making and focus on family and community priorities reflects both cultural and economic differences between first world, western nations and non-western, developing countries. The findings can serve as a valuable starting point for the development of a globally applicable measurement for the quality of care.

